# Impact of *Helicobacter pylori* Infection Status on SEPTIN9 and SDC2 Methylation Levels and Their Association with Clinical Outcomes in Elderly Patients with Gastric Mucosal Intestinal Metaplasia

**DOI:** 10.5152/tjg.2026.26162

**Published:** 2026-07-21

**Authors:** Zhuwen Yu, Haoxiang Ni, Xiaojuan Chen, Kunting Xiao

**Affiliations:** 1 Department of Gastroenterology, The First Affiliated Hospital of Soochow University, Suzhou, China; 2 Department of Geriatrics, The First Affiliated Hospital of Soochow University, Suzhou, China

**Keywords:** DNA methylation, intestinal metaplasia, *Helicobacter pylori*, risk stratification, SDC2, SEPTIN9

## Abstract

**Background/Aims:**

The impact of dynamic *Helicobacter pylori* (Hp) infection status on DNA methylation and progression risk in elderly patients with gastric intestinal metaplasia (IM) remains unclear. This study evaluated the associations of Hp infection status with *SEPTIN9* and *SDC2* methylation levels and whether baseline methylation markers were associated with subsequent clinical outcomes.

**Materials and Methods:**

This single-center retrospective cohort study enrolled 200 older adults with IM. Hp infection status was dynamically defined over follow-up as persistent infection, successful eradication, or sustained negative infection. Baseline *SEPTIN9* and *SDC2* methylation levels were quantified in formalin-fixed, paraffin-embedded tissue samples using quantitative methylation-specific polymerase chain reaction (qMSP). Kaplan–Meier analysis and Cox proportional hazards regression were used to evaluate progression risk. Predictive performance was assessed using Harrell’s C-index and time-dependent receiver operating characteristic analysis. Because methylation was measured only at baseline, the analyses were designed to evaluate baseline methylation markers rather than dynamic methylation changes over time.

**Results:**

During a median follow-up of 3.86 years, 38 patients (19.0%) experienced disease progression. Persistent Hp infection was associated with higher *SEPTIN9* and *SDC2* methylation levels (*P* < .01). Combined double-positive methylation status was associated with an increased risk of IM progression (adjusted hazard ratio [HR] = 3.12, 95% CI: 1.53-6.38, *P* = .002), with the strongest association observed among patients with persistent Hp infection. Incorporation of the combined methylation indicator improved model discrimination (C-index 0.75 vs. 0.68) and the 5-year area under the curve (0.77 vs. 0.69).

**Conclusion:**

Baseline *SEPTIN9* and *SDC2* methylation levels differed according to the Hp infection status, with the highest levels observed among patients with persistent Hp infection. Baseline combined methylation status was associated with an increased risk of IM progression and may provide incremental value for risk stratification. These findings should be interpreted as baseline associations.

**Main Points**

- Persistent *Helicobacter pylori* (Hp) infection is associated with higher *SEPTIN9* and *SDC2* methylation levels in older adults with intestinal metaplasia (IM), whereas successful eradication is associated with moderate methylation levels.
- Baseline double-positive *SEPTIN9* and *SDC2* methylation is associated with an increased risk of IM progression, with the strongest association observed among patients with persistent Hp infection.
- Incorporating the combined *SEPTIN9* and *SDC2* methylation marker may improve prediction of IM progression and enhance effective individualized risk stratification in older adults, although this reflects baseline risk stratification rather than dynamic methylation monitoring.

## Introduction

Gastric mucosal intestinal metaplasia (IM) is a well-established premalignant lesion in the Correa cascade of gastric carcinogenesis.^1^ The progression from chronic gastritis to atrophic gastritis, IM, dysplasia, and ultimately gastric cancer is a multistep and multifactorial process influenced by environmental, microbial, and host factors.^2^ Among these, *Helicobacter pylori* (Hp) infection plays a central etiological role by inducing chronic inflammation, oxidative stress, and epigenetic alterations within the gastric mucosa.^3^ Although Hp eradication reduces the risk of gastric cancer, many patients with established IM continue to exhibit persistent molecular abnormalities, highlighting the need for reliable biomarkers to refine risk stratification.

DNA methylation is a key epigenetic mechanism involved in inflammation-associated carcinogenesis.^4^ Aberrant promoter methylation of tumor-related genes may occur early in gastric mucosal transformation and accumulate during disease progression.^5^ *SEPTIN9* and *SDC2* are genes frequently reported to undergo cancer-related hypermethylation and have been investigated as noninvasive biomarkers in gastrointestinal malignancies.^6^ However, their methylation patterns in premalignant gastric lesions, particularly in relation to follow-up-defined Hp infection status, remain insufficiently characterized.

Older adults are at increased risk for gastric neoplastic progression because of longer cumulative exposure to inflammatory stimuli and age-associated epigenetic drift.^7^^,^^8^ Understanding whether persistent Hp infection modulates *SEPTIN9* and *SDC2* methylation levels and whether combined methylation status improves prediction of clinical outcomes is of substantial clinical importance.

Therefore, this study investigated the association between follow-up-defined Hp infection status and methylation levels of *SEPTIN9* and *SDC2* in older adults with gastric IM and evaluated the incremental predictive value of combined methylation status for risk stratification of disease progression.

## Materials and Methods

### Study Design and Participants

This was a single-center retrospective cohort study with longitudinal follow-up. Older adults who underwent gastroscopy at The First Affiliated Hospital of Soochow University and were pathologically diagnosed with IM were consecutively enrolled. Patients were retrospectively identified at baseline, and clinical outcomes were subsequently followed until December 2025. The study period spanned from January 2020 to December 2025. Given the retrospective design, the study may be subject to inherent selection bias.

The inclusion criteria were as follows: (1) age ≥60 years; (2) histopathological confirmation of IM based on baseline endoscopic biopsy; (3) availability of baseline formalin-fixed, paraffin-embedded (FFPE) gastric mucosal tissue specimens suitable for DNA methylation analysis; and (4) at least 1 follow-up endoscopic examination or clearly documented clinical outcomes during follow-up.

The exclusion criteria were as follows: (1) gastric cancer or high-grade intraepithelial neoplasia (HGIN) at baseline; (2) low-grade intraepithelial neoplasia (LGIN) at baseline; (3) history of prior gastrectomy; and (4) incomplete clinical data or unavailable tissue specimens. Therefore, all included patients had histopathologically confirmed IM without dysplasia at baseline, and any occurrence of LGIN, HGIN, or gastric cancer was considered to have developed during follow-up.

A total of 200 patients were included in the final analysis. Ethical approval was obtained from the Ethics Committee of The First Affiliated Hospital of Soochow University on January 5, 2020 (Approval No. 2026-807). Patient data were retrospectively extracted from existing medical records and analyzed longitudinally for outcome assessment. The requirement for informed consent was waived because of the retrospective design and use of de-identified data. No artificial intelligence and/or large language model (LLM) tools were used in the preparation of this manuscript.

### Sample Size and Statistical Power Assessment

This study was designed as a retrospective cohort study, and the sample size was determined by the number of eligible patients identified during the study period. To evaluate model stability, the commonly accepted events-per-variable (EPV) principle for survival analysis was followed, which recommends approximately 10 outcome events per variable included in a Cox proportional hazards model. A total of 200 patients were included in the analysis, and 38 experienced disease progression during follow-up. The number of covariates in the primary multivariable Cox model was limited to satisfy the EPV requirement. However, despite meeting the EPV criterion, the relatively limited number of outcome events may still affect the robustness and generalizability of the model.

### Definition and Stratification of *Helicobacter pylori* Infection Status

Hp infection status was determined using a combination of histopathology, rapid urease test (RUT), and/or 13C urea breath test (UBT). Although multiple diagnostic methods were used, potential misclassification cannot be entirely excluded.

For histological examination, biopsy specimens were obtained from the gastric antrum and corpus during endoscopy. Biopsy sampling was performed in accordance with established clinical guidelines, specifically the Updated Sydney System, using predefined anatomical sites to ensure standardized and reproducible assessment of Hp infection. At least 2 biopsy specimens were obtained from the gastric antrum (including the lesser and greater curvature within 2-3 cm of the pylorus) and at least 2 from the gastric corpus (including the lesser and greater curvature of the mid-body). Tissue sections were stained with hematoxylin and eosin and modified Giemsa stain (Leica Biosystems, Wetzlar, Germany). Slides were independently evaluated by experienced gastrointestinal pathologists. The presence of characteristic curved or spiral-shaped bacilli in any biopsy specimen was considered indicative of Hp positivity.

The RUT was performed immediately after obtaining antral mucosal biopsy specimens during endoscopy. Samples were placed into a commercial urease detection kit (Pronto Dry® Rapid Urease Test, Medical Instruments Corporation, Solothurn, Switzerland), and color change was observed at room temperature according to the manufacturer’s instructions. A color shift from yellow to red within the specified time was considered a positive result.

The 13C UBT was performed using a HUBT-20P infrared spectrometer with a corresponding 13C-UBT kit (Headway Bio-Sci & Tech Co., Shenzhen, China). After overnight fasting, participants ingested 13C-labeled urea, and breath samples were collected for analysis. A delta-over-baseline value ≥4‰ was defined as Hp positive. To minimize false-negative results, proton pump inhibitors (PPIs) were discontinued for at least 1 week before testing, and antibiotics were withheld according to routine clinical practice guidelines. In cases of discordant results among different diagnostic methods, histopathology or the 13C UBT result was prioritized for final determination.

During follow-up, patients underwent repeat endoscopy and/or Hp-related testing according to clinical indications, typically every 1-2 years, in line with routine clinical practice. Based on Hp test results and eradication therapy status during follow-up, patients were classified into 3 groups: (1) persistent positive group (Hp remained positive during follow-up), (2) successful eradication group (baseline positive with subsequent negative conversion after eradication therapy), and (3) sustained negative group (negative at baseline and throughout follow-up). In patients who received eradication therapy, *H. pylori* eradication was confirmed based on follow-up testing, typically at least 4 weeks after completion of treatment, using 13C-UBT and/or histological assessment in accordance with routine clinical practice.

### Collection of Clinical, Endoscopic, and Histopathological Data

Baseline demographic and clinical data were retrieved from the hospital electronic medical record system. Variables collected included age, sex, body mass index (BMI), smoking history, alcohol consumption, presence of diabetes mellitus and cardiovascular disease, and use of PPIs. Information on family history of gastric cancer in first-degree relatives was collected from medical records and included as a covariate in the analysis. Smoking and alcohol history were obtained from medical records and admission interviews. Current and former smokers were combined and categorized as “current/former smokers.”

Endoscopic examinations were performed according to standard gastroscopy procedures. Mucosal biopsy specimens were obtained according with the Updated Sydney System from predefined anatomical sites, including the gastric antrum and corpus, to ensure standardized sampling for histopathological evaluation. The extent of IM was classified based on pathological reports as focal (single site) or multifocal (≥2 anatomical sites). Multifocal IM was used as a surrogate for extensive IM, reflecting the spatial extent of IM. Incomplete-type IM was diagnosed according to established histopathological criteria, including the proportion of goblet cells and epithelial differentiation patterns.

Gastric mucosal atrophy and inflammatory activity were graded according to the Updated Sydney System, with scores ranging from 0 to 3 (0 = none, 1 = mild, 2 = moderate, and 3 = severe). The Operative Link on Gastric Intestinal Metaplasia (OLGIM) staging system was applied based on the distribution and severity of IM in antral and corpus biopsies. For statistical analysis, OLGIM stages were further dichotomized as <stage 2 and ≥stage 2.

All histopathological slides were independently evaluated by experienced gastrointestinal pathologists blinded to patients’ follow-up outcomes and methylation test results to minimize observer bias.

### Follow-Up and Definition of Outcomes

Follow-up duration was defined as the time interval from the date of baseline IM diagnosis to disease progression, last follow-up visit, or the end of the study period, whichever occurred first. Follow-up was conducted according to routine clinical practice, typically at intervals of 1-2 years. Outcome events (LGIN, HGIN, and gastric cancer) were confirmed histologically based on endoscopic biopsy or resection specimens obtained during follow-up. Outcome adjudication was performed blinded to methylation test results to minimize observer bias. The primary endpoint was defined as progression of IM, operationalized as the development of LGIN, HGIN, or gastric cancer during follow-up. This composite endpoint was used to capture the full spectrum of neoplastic progression along the gastric carcinogenesis cascade and to improve statistical power, given the relatively limited number of advanced events.

### DNA Extraction from Formalin-Fixed Paraffin-Embedded Tissues and Detection of SEPTIN9 and SDC2 Methylation

Serial 5-10 μm sections were obtained from baseline gastric mucosal FFPE tissues. After deparaffinization with xylene and rehydration through graded ethanol, genomic DNA was extracted using the QIAamp DNA FFPE Tissue Kit (Qiagen, Hilden, Germany) according to the manufacturer’s instructions. DNA concentration and purity were measured using a NanoDrop 2000 spectrophotometer (Thermo Fisher Scientific, Wilmington, DE, USA). DNA was then subjected to bisulfite conversion using the EZ DNA Methylation-Gold Kit (Zymo Research, Irvine, CA, USA), which converts unmethylated cytosines to uracils, enabling subsequent methylation-specific amplification. Converted DNA was either immediately used for polymerase chain reaction (PCR) analysis or stored at −20°C until further processing.

Methylation levels of SEPTIN9 and SDC2 were quantified using quantitative methylation-specific PCR. PCR reactions were performed on an ABI 7500 Real-Time PCR System (Applied Biosystems, Foster City, CA, USA) using TaqMan Universal PCR Master Mix (Applied Biosystems). β-actin was included in each reaction as an internal reference gene for quality control.

Methylation levels are expressed as the percentage of methylated reference (PMR) values and log-transformed (logPMR) to meet statistical assumptions. *SEPTIN9* and *SDC2* methylation indices were further standardized using *Z*-score transformation, calculated as: *Z* = (*X* − *μ*)/*σ* where *X* represents the individual logPMR value and *μ* and *σ* denote the mean and SD for each marker, respectively. A *Z*-score > 0 was considered relatively high methylation (positive), whereas a *Z*-score ≤ 0 was considered relatively low methylation (negative). A combined methylation score was generated by summing the standardized *Z*-scores of *SEPTIN9* and *SDC2*. Because methylation was measured only in baseline FFPE tissues, no longitudinal methylation assessment was available. Accordingly, subsequent analyses evaluated associations between baseline methylation measures and future clinical outcomes.

### Definition of Combined Methylation Status and Construction of the Predictive Model

Based on the combined *SEPTIN9* and *SDC2* methylation score, patients were categorized into 3 groups: double negative, single positive, and double positive. These categories were derived using standardized *Z*-score thresholds and reflected relative methylation levels within the study cohort. They were used in Kaplan–Meier survival analysis and Cox proportional hazards regression to evaluate the association between combined methylation status and the risk of IM.

For predictive modeling, a base clinical model was first constructed, including age, sex, smoking status, OLGIM stage, and Hp infection status. The combined methylation indicator was then added to the base model to generate an extended model (combined model). Given the limited number of progression events, the number of covariates in the multivariable model was restricted in accordance with the EPV principle to reduce the risk of overfitting. Model discrimination was assessed using Harrell’s concordance index (C-index). Time-dependent receiver operating characteristic (ROC) curve analysis was performed to estimate the area under the curve (AUC) at 5 years. These analyses were used to evaluate model discrimination; however, no external validation was performed, and model calibration was not formally assessed.

Based on the multivariable Cox proportional hazards model in the extended model, a linear predictor was calculated for each patient as an individual risk score. Patients were then stratified into low-, intermediate-, and high-risk groups according to tertiles of the risk score distribution.

### Statistical Analysis

All statistical analyses were performed using SPSS version 26.0 (IBM SPSS Corp.; Armonk, NY, USA). Continuous variables are presented as mean ± SD. Normality was assessed using the Shapiro–Wilk test before analysis. Normally distributed continuous variables were compared between groups using 1-way analysis of variance. Categorical variables are presented as counts and percentages and compared using the *χ*^2^ test.

Multivariable linear regression analysis was used to examine the association between follow-up-defined Hp infection status and methylation levels. All statistical tests were 2-sided, and a *P* value < .05 was considered statistically significant. Because methylation data were available only at baseline, no longitudinal or repeated-measures analyses of methylation dynamics were performed.

## Results

### Baseline Characteristics of the Study Population

A total of 200 older adults with gastric mucosal IM were included and categorized into 3 groups based on follow-up-defined Hp infection status during follow-up: persistent positive (n = 72), successful eradication (n = 68), and sustained negative (n = 60). Baseline characteristics were generally comparable across the 3 groups, with no significant differences in age, sex distribution, BMI, family history of gastric cancer, or the prevalence of diabetes mellitus and cardiovascular disease (all *P* > .05).

However, compared with the sustained negative group, patients in the persistent positive group had a higher proportion of current/former smokers, higher gastric mucosal inflammatory activity scores, and a greater proportion of OLGIM stage ≥ 2 (all *P* < .05). The successful eradication group showed intermediate findings between the persistent positive and sustained negative groups for multifocal IM and atrophy scores. In contrast, PPI use did not differ significantly among the 3 groups (*P* > .05) (Table 1).

### Association Between Follow-Up–Defined *Helicobacter pylori* Status and SEPTIN9/SDC2 Methylation Levels

Quantitative methylation-specific PCR analysis of baseline gastric mucosal FFPE tissues demonstrated significant differences in *SEPTIN9* and *SDC2* methylation levels among the 3 dynamic Hp infection status groups (Table 2).

For both *SEPTIN9* and *SDC2*, methylation levels showed a stepwise decrease across groups, with the highest levels observed in the persistent positive group, intermediate levels in the successful eradication group, and the lowest levels in the sustained negative group (all *P* < .001). A similar pattern was observed for the combined methylation score.

To further assess whether Hp infection status was independently associated with methylation levels, multivariable linear regression analysis was performed after adjusting for age, sex, smoking status, and OLGIM stage (Table 3). Persistent Hp positivity remained significantly associated with higher *SEPTIN9* and *SDC2* methylation levels compared with the sustained negative group (both *P* < .01). Successful eradication status was also associated with higher methylation levels, although the magnitude of this association was weaker. These findings indicate a graded association between Hp infection status and methylation levels. However, because methylation was assessed only at baseline, the results should be interpreted as cross-sectional associations rather than reflecting dynamic changes over time.

### Combined Methylation Status and Risk of Intestinal Metaplasia Progression

#### Kaplan–Meier Survival Analysis:

The median follow-up duration was 3.86 years (interquartile range: 1.24-5.72 years). During follow-up, 38 patients (19.0%) experienced disease progression, including 20 cases of LGIN, 12 cases of HGIN, and 6 cases of gastric cancer, all of which were newly diagnosed. In the overall cohort, patients with progression had higher* SEPTIN9* and *SDC2* methylation levels, as well as higher combined methylation scores, than those without progression. Stratified analyses showed that this difference was most pronounced in the Hp-positive subgroup, whereas a similar but nonsignificant trend was observed in the Hp-eradicated subgroup. No significant differences were identified in the Hp-negative subgroup (Table 4). Based on the combined *SEPTIN9* and *SDC2* methylation score, patients were categorized into 3 groups: double negative (n = 74), single positive (n = 68), and double positive (n = 58). Kaplan–Meier survival analysis demonstrated significant differences in progression-free survival among the 3 combined methylation groups (log-rank *χ*^2^ = 18.62, *P* < .001). A clear stepwise increase in cumulative progression risk was observed across the groups, with the highest risk in the double-positive group and the lowest risk in the double-negative group (Figure 1). These findings support an association between combined methylation status and IM progression risk.

#### Cox Proportional Hazards Regression Analysis:

To further evaluate the independent association between combined methylation status and IM progression, Cox proportional hazards regression analysis was performed. In univariable analysis, double-positive combined methylation status was significantly associated with an increased risk of progression compared with the double-negative group (HR = 3.94, 95% CI: 2.02-7.70, *P* < .001), whereas the single-positive group did not reach statistical significance (HR = 1.82, 95% CI: 0.88-3.75, *P* = .10).

In the multivariable Cox model adjusting for age, sex, smoking status, OLGIM stage, and Hp infection status, double-positive methylation status remained an independent predictor of IM progression (HR = 3.12, 95% CI: 1.53-6.38, *P* = .002). Additionally, OLGIM stage ≥ 2 was significantly associated with increased progression risk (HR = 2.01, 95% CI: 1.05-3.84, *P* = .03), whereas persistent Hp positivity was not independently associated with progression after adjustment. Given that methylation status was measured only at baseline, this result should be interpreted as an association between baseline methylation status and subsequent progression risk.

To further assess the robustness of the findings, a sensitivity analysis was performed using OLGIM stage ≥ 3 as the cutoff. The association between higher OLGIM stage and increased risk of IM progression remained directionally consistent with the primary analysis. However, this analysis was limited by reduced statistical power because of the smaller number of patients in the OLGIM ≥ 3 subgroup.

#### Interaction and Stratified Analysis by *Helicobacter pylori* Infection Status:

To explore whether Hp infection status modified the association between combined methylation and progression risk, an interaction term was included in the multivariable Cox model. A statistically significant interaction was observed (Pinteraction = 0.03), indicating that the prognostic effect of methylation status varied according to Hp infection status.

Stratified analyses further demonstrated that, among patients with persistent Hp infection, double-positive combined methylation status was significantly associated with an increased risk of IM progression (HR = 4.26, 95% CI: 1.88-9.64). However, subgroup analyses may be limited by reduced sample size. In contrast, this association was attenuated and not statistically significant among patients with sustained negative Hp status (HR = 1.74, 95% CI: 0.62-4.88). To further illustrate this interaction, Kaplan–Meier curves stratified by Hp infection status were constructed (Figure 2), demonstrating greater separation of progression-free survival curves across methylation groups among patients with persistent Hp infection. These findings suggest that the prognostic association of combined *SEPTIN9* and *SDC2* methylation is more significant in the presence of persistent Hp infection. However, given the baseline measurement of methylation and the limited sample size in subgroup analyses, these findings should be interpreted with caution.

### Predictive Performance of the Combined Methylation Indicator

#### Comparison of Model Discrimination Between the Base and Combined Models:

To evaluate the incremental predictive value of the combined *SEPTIN9* and *SDC2* methylation indicator for IM progression, a base clinical model (including age, sex, smoking status, OLGIM stage, and Hp infection status) was compared with an extended model incorporating the combined methylation indicator. The C-index of the base model was 0.68 (95% CI: 0.60-0.76), which increased to 0.75 (95% CI: 0.68-0.82) after inclusion of the combined methylation indicator, indicating improved discriminatory performance (ΔC-index = 0.07). However, the magnitude of this improvement was modest, and should be interpreted with caution, particularly in the absence of external validation.

#### Time-Dependent Predictive Performance:

Time-dependent ROC curve analysis further demonstrated improved predictive performance of the combined model compared with the base model at the 5-year follow-up. The 5-year AUC was 0.77 (95% CI: 0.70-0.84) for the combined model and 0.69 (95% CI: 0.61-0.77) for the base model (Figure 3). These results indicate that incorporating baseline methylation markers enhances risk prediction for IM progression.

#### Clinical Implications of Risk Stratification Performance:

Based on tertiles of the individual risk scores derived from the combined model, patients were categorized into low-, intermediate-, and high-risk groups. Kaplan–Meier survival analysis demonstrated significant differences in progression-free survival among these groups (log-rank *P* < .001), with a clear gradient in progression risk, highest in the high-risk group and lowest risk in the low-risk group (Figure 4). These findings suggest that the model can effectively stratify patients according to progression risk. However, prospective validation is required before clinical application.

## Discussion

In this retrospective cohort study of elderly patients with gastric IM, 3 principal findings were demonstrated. First, baseline *SEPTIN9* and *SDC2* methylation levels differed across groups defined by subsequent Hp infection status, with the highest levels observed among patients with persistent Hp infection and intermediate levels among those with successful eradication. Second, baseline double-positive combined methylation status was independently associated with an increased risk of IM progression in the overall cohort after adjustment for conventional risk factors, including OLGIM stage and Hp infection. However, this association differed according to Hp status, indicating potential effect modification rather than a uniform predictive effect across all subgroups. Third, incorporation of the combined methylation indicator improved model discrimination and mid-term predictive performance. Overall, these findings suggest associations between Hp-related factors, epigenetic alterations, and disease progression, rather than implying causal or fully predictive relationships.

Epidemiological studies have demonstrated that IM is a key premalignant lesion associated with an increased risk of gastric cancer.^9^ Hp infection is a well-established etiological factor, driving carcinogenesis through inflammation and oxidative stress.^10^^,^^11^ Mechanistic studies have demonstrated that persistent Hp colonization induces reactive oxygen species, cytokine activation, and epithelial genomic instability.^10^^,^^12^ Increasing evidence suggests that inflammation-induced epigenetic alterations, particularly DNA methylation, may represent an intermediate mechanism linking Hp infection to neoplastic transformation.

DNA methylation is a critical regulator in inflammation-associated carcinogenesis.^13^^,^^14^ Aberrant promoter methylation of tumor-suppressor genes has been observed in Hp-infected gastric mucosa before the development of overt dysplasia.^15^^,^^16^ Chronic inflammatory signaling may activate DNA methyltransferases, leading to stable epigenetic silencing.^17^ Importantly, although Hp eradication reduces methylation levels, certain changes may persist, particularly in atrophic or metaplastic mucosa.^18^ However, methylation was assessed only at baseline in this study, with no longitudinal measurements available; therefore, the observed differences across Hp status groups should be interpreted as baseline associations rather than dynamic epigenetic changes. Consistent with this, baseline *SEPTIN9* and *SDC2* methylation levels varied by Hp status, being highest in the persistent positive group, intermediate in the eradicated group, and lowest in the sustained negative group. These findings support an inflammation–epigenetic axis and suggest that prolonged Hp exposure may contribute to sustained epigenetic alterations. However, because of the observational nature of this study, causal inference cannot be established. In addition, age-associated epigenetic drift in older adults may further contribute to disease susceptibility.^19^

Stratified analyses further demonstrated that the association between combined methylation status and progression risk varied according to Hp status. The prognostic effect was most pronounced in the persistent Hp-positive subgroup, whereas no significant association was observed in the Hp-negative subgroup. In patients with successful eradication, a similar but nonsignificant trend was observed. Importantly, although a composite endpoint was used to define progression, the numbers of individual endpoint components (LGIN, HGIN, and gastric cancer) were reported separately, which partially improves interpretability despite the inherent heterogeneity in clinical severity. This pattern is biologically plausible, as persistent infection may maintain an inflammatory microenvironment that amplifies methylation-driven carcinogenic pathways, whereas eradication may attenuate, but not fully reverse, these alterations. The lack of significance in the eradicated subgroup may also reflect limited statistical power.

Although persistent Hp infection was associated with higher methylation levels, it was not an independent predictor of IM progression after multivariable adjustment, suggesting that its effect may be mediated through downstream molecular alterations rather than acting as a direct driver. In addition, no clear short-term protective effect of Hp eradication on IM progression was observed during the median follow-up of approximately 3 years, possibly reflecting the slow and partially irreversible nature of epigenetic alterations in metaplastic mucosa.

*SEPTIN9* and *SDC2* methylation have been extensively studied as biomarkers in colorectal cancers, mainly for early detection.^6^^,^^20^ However, their roles in premalignant gastric lesions are limited. The study suggests that combined methylation status may provide additional prognostic value beyond conventional histopathological staging. The observed association likely reflects underlying molecular alterations associated with disease progression. Importantly, although OLGIM stage remained significant, persistent Hp infection did not retain independent significance in multivariable analysis, suggesting that methylation markers may partially capture downstream molecular consequences of Hp-induced injury.

From a clinical perspective, risk stratification in IM remains challenging. Current guidelines rely primarily on endoscopic and histological staging systems such as OLGIM.^21^ In the study, addition of the combined methylation indicator to a base clinical model improved the C-index from 0.68 to 0.75 and increased the 5-year time-dependent AUC from 0.69 to 0.77. However, the magnitude of improvement in discrimination was modest and should be interpreted with caution, particularly in the absence of external validation. These findings suggest potential incremental predictive value; however, the absence of external validation and formal calibration assessment limits the generalizability and robustness of the model. Moreover, tertile-based risk categorization derived from the combined model effectively separated patients into distinct prognostic groups, supporting its potential utility for individualized surveillance intervals.

Several limitations merit consideration. First, this was a single-center retrospective study, which may limit generalizability. Second, methylation was assessed only at baseline, with no longitudinal measurements available, precluding evaluation of temporal epigenetic dynamics and limiting causal inference. Third, although model complexity was constrained by the EPV principle, the relatively small number of progression events may compromise robustness and increase the risk of overfitting. Fourth, the use of a composite endpoint may introduce clinical heterogeneity, although individual components were reported separately. Fifth, methylation categories were derived from cohort-specific *Z*-score thresholds rather than externally validated cutoffs, potentially limiting reproducibility. Sixth, the model was evaluated using internal metrics only, without external validation or calibration assessment. Seventh, unmeasured confounders, including dietary factors, vitamin status, and bile reflux, may have introduced residual bias. In addition, other important confounders, such as noneradication antibiotic exposure and microbiome-related variables, were not consistently available and were therefore not included, which may have influenced methylation patterns and introduced residual confounding. Finally, no mechanistic experiments were performed; thus, the observed associations should not be interpreted as causal. Future multicenter prospective studies incorporating serial methylation measurements, detailed exposure data, and microbiome analyses are warranted.

In conclusion, baseline *SEPTIN9* and *SDC2* methylation levels differ according to Hp infection status, with higher levels observed in patients with persistent Hp infection. Combined methylation status is associated with an increased risk of disease progression and may improve risk stratification when integrated into clinical models. However, given the study design and limited number of events, these findings should be interpreted with caution and require validation in larger, multicenter prospective cohorts.

## Figures and Tables

**Figure 1.**
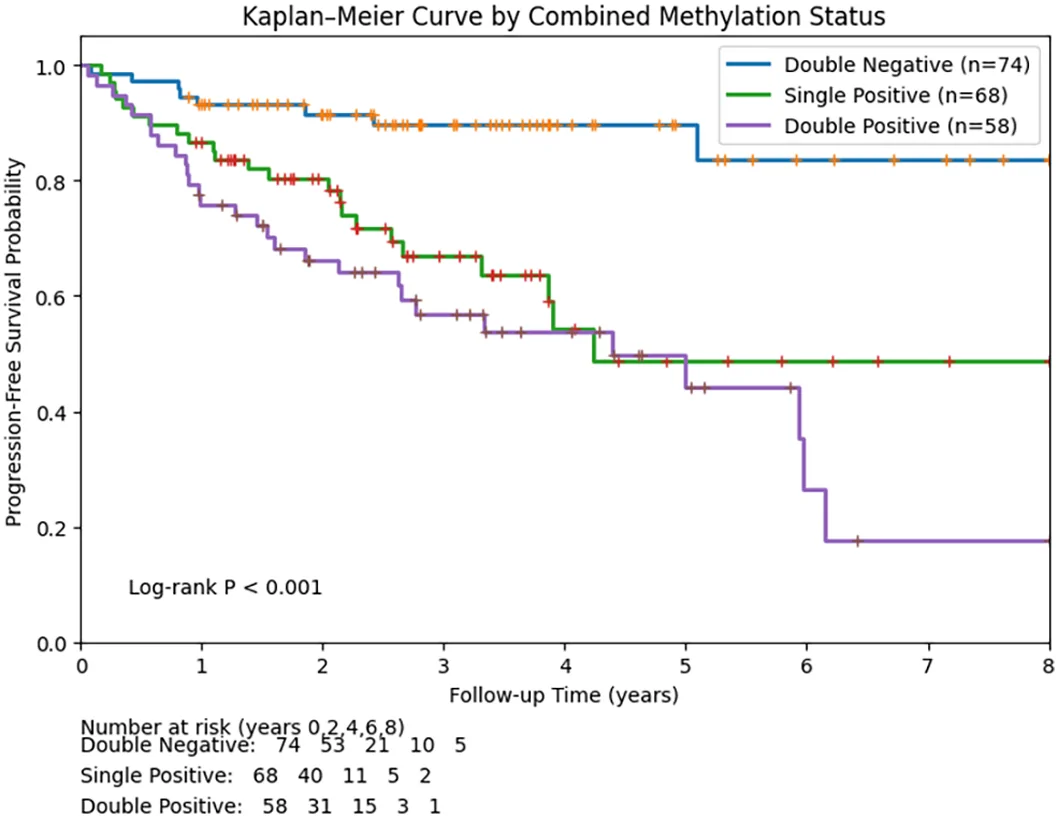
Kaplan–Meier progression-free survival curves according to different combined methylation status groups. The “+” symbols indicate censored individuals.

**Figure 2.**
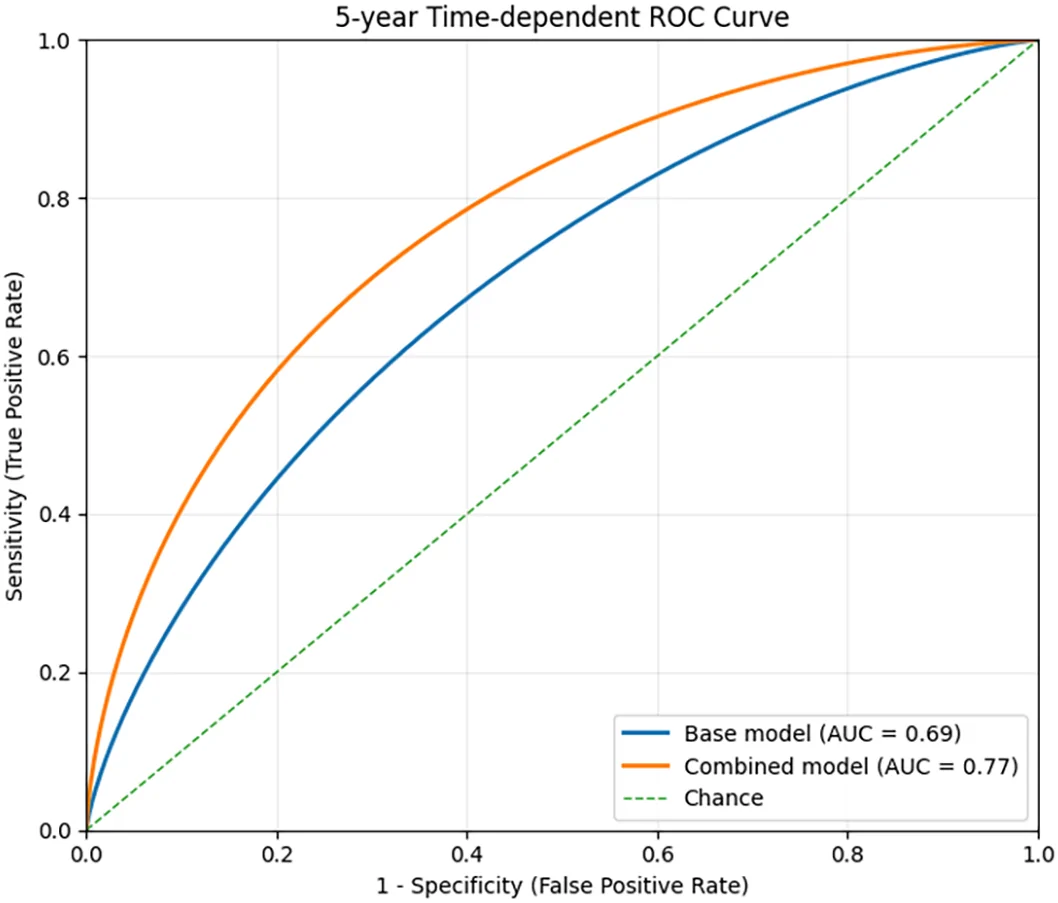
Five-year time-dependent receiver operating characteristic curves comparing the predictive performance of the base model and the combined model.

**Figure 3.**
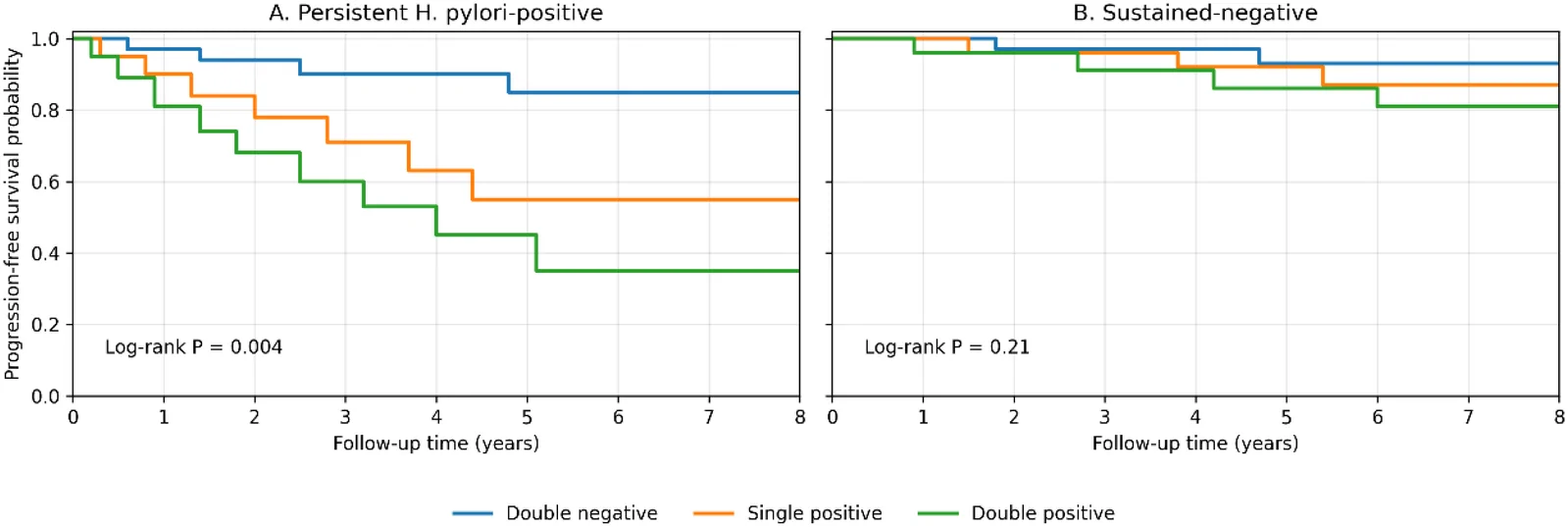
Kaplan–Meier curves for progression-free survival according to combined methylation status stratified by *Helicobacter pylori* infection status.

**Figure 4.**
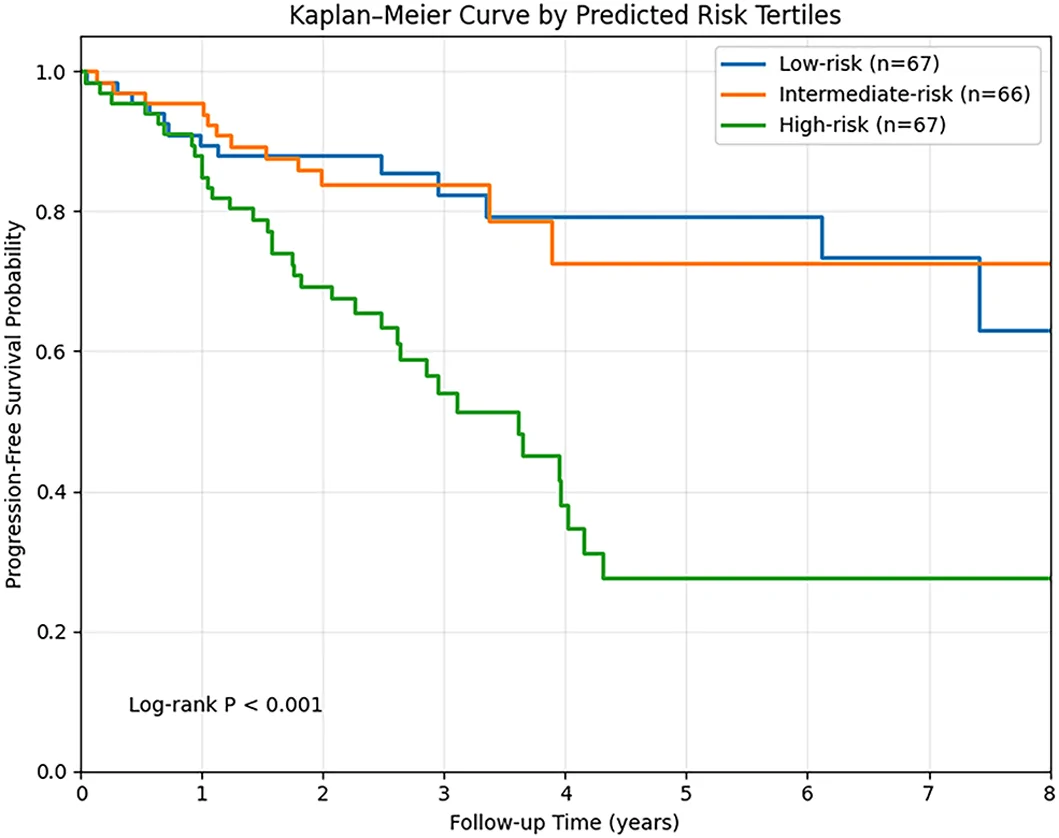
Kaplan–Meier progression-free survival curves stratified by tertiles of predicted risk based on the combined model.

**Table 1.** Baseline Characteristics of the Study Population According to Dynamic *Helicobacter pylori* Infection Status

Variable	Overall (N = 200)	Persistent Positive (n = 72)	Successful Eradication (n = 68)	Sustained Negative (n = 60)	Statistic	*P*
Age (years), mean ± SD	68.92 ± 6.81	69.11 ± 6.74	68.72 ± 6.63	68.88 ± 7.12	*F* = 0.058	.944
Male, n (%)	118 (59.00)	45 (62.50)	40 (58.82)	33 (55.00)	*χ*^2^ = 0.762	.683
BMI (kg/m^2^), mean ± SD	24.08 ± 3.21	23.91 ± 3.18	24.22 ± 3.27	24.13 ± 3.19	*F* = 0.173	.842
Family history of gastric cancer, n (%)	26 (13.00)	12 (16.67)	9 (13.24)	5 (8.33)	*χ*^2^ = 2.215	.331
Smoking (current/former), n (%)	77 (38.50)	36 (50.00)	25 (36.76)	16 (26.67)	*χ*^2^ = 7.656	.022
Alcohol use (current/former), n (%)	52 (26.00)	21 (29.17)	19 (27.94)	12 (20.00)	*χ*^2^ = 1.631	.442
Diabetes mellitus, n (%)	58 (29.00)	23 (31.94)	20 (29.41)	15 (25.00)	*χ*^2^ = 2.531	.282
Cardiovascular disease, n (%)	96 (48.00)	36 (50.00)	33 (48.53)	27 (45.00)	*χ*^2^ = 0.339	.844
PPI use (≥4 weeks), n (%)	74 (37.00)	28 (38.89)	26 (38.24)	20 (33.33)	*χ*^2^ = 0.501	.779
Multifocal IM (≥2 sites), n (%)	112 (56.00)	45 (62.50)	39 (57.35)	28 (46.67)	*χ*^2^ = 3.406	.182
Incomplete-type IM, n (%)	86 (43.00)	36 (50.00)	30 (44.12)	20 (33.33)	*χ*^2^ = 3.762	.153
Atrophy score, mean ± SD	1.31 ± 0.71	1.36 ± 0.69	1.33 ± 0.73	1.23 ± 0.72	*F* = 0.582	.560
Activity score, mean ± SD	1.09 ± 0.83	1.58 ± 0.77	1.06 ± 0.81	0.63 ± 0.59	*F* = 27.617	<.001
OLGIM stage ≥ 2, n (%)	94 (47.00)	41 (56.94)	33 (48.53)	20 (33.33)	*χ*^2^ = 7.421	.025
Recommended follow-up interval 1-2 years, n (%)	126 (63.00)	49 (68.06)	43 (63.24)	34 (56.67)	*χ*^2^ = 1.824	.402

Recommended follow-up interval reflects the suggested surveillance interval based on clinical guidelines and does not represent actual follow-up timing. Family history of gastric cancer was defined as a history of gastric cancer in first-degree relatives.

BMI, body mass index; Hp, *Helicobacter pylori*; IM, intestinal metaplasia; OLGIM, Operative Link on Gastric Intestinal Metaplasia; PPI, proton pump inhibitor.

**Table 2.** Comparison of *SEPTIN9* and *SDC2* Methylation Levels Among Different Dynamic Hp Infection Groups

Variable	Overall (N = 200)	Persistent Positive (n = 72)	Successful Eradication (n = 68)	Sustained Negative (n = 60)	Statistic	*P*
*SEPTIN9* methylation index (logPMR), mean ± SD	0.84 ± 0.52	1.12 ± 0.49	0.86 ± 0.47	0.54 ± 0.39	*F* = 26.567	<.001
*SDC2* methylation index (logPMR), mean ± SD	0.79 ± 0.48	1.03 ± 0.46	0.81 ± 0.45	0.55 ± 0.36	*F* = 20.497	<.001
Combined methylation score (sum of *Z*-scores), mean ± SD	0.00 ± 1.36	0.63 ± 1.22	0.05 ± 1.28	−0.78 ± 1.11	*F* = 22.230	<.001

Hp, *Helicobacter pylori*; PMR, percentage of methylated reference.

**Table 3.** Multivariable Linear Regression Analysis of the Association Between Follow-up-Defined Hp Infection Status and Methylation Levels

Variable	β Coefficient	Standard Error	*t*	*P*
Dependent variable: *SEPTIN9* logPMR				
Persistent positive Hp (vs. sustained negative)	0.42	0.09	4.67	<.001
Successful eradication (vs. sustained negative)	0.21	0.08	2.63	.009
Age	0.01	0.01	0.89	.37
Male	0.05	0.07	0.71	.48
Smoking (current/former)	0.11	0.06	1.83	.07
OLGIM stage ≥ 2	0.18	0.07	2.57	.01
Dependent variable: *SDC2* logPMR				
Persistent positive Hp (vs. sustained negative)	0.36	0.08	4.21	<.001
Successful eradication (vs. sustained negative)	0.17	0.07	2.31	.02
Age	0.01	0.01	0.74	.46
Male	0.03	0.06	0.52	.60
Smoking (current/former)	0.09	0.05	1.72	.09
OLGIM stage ≥ 2	0.15	0.06	2.42	.02

*β*, regression coefficient; Hp, *Helicobacter pylori*; OLGIM, Operative Link on Gastric Intestinal Metaplasia; PMR, percentage of methylated reference.

**Table 4.** Comparison of Methylation Scores Between Patients with and Without Progression, Overall and Stratified by *Helicobacter pylori* Status

Variable	No Progression	Progression	*P*
Overall cohort	n = 162	n = 38	
*SEPTIN9* (logPMR)	0.76 ± 0.47	1.18 ± 0.49	<.001
*SDC2* (logPMR)	0.71 ± 0.43	1.11 ± 0.44	<.001
Combined methylation score (*Z*-score)	−0.18 ± 1.21	0.79 ± 1.18	<.001
Sustained negative	n = 52	n = 8	
*SEPTIN9* (logPMR)	0.50 ± 0.35	0.69 ± 0.38	.163
*SDC2* (logPMR)	0.51 ± 0.33	0.67 ± 0.35	.210
Combined methylation score (*Z*-score)	−0.86 ± 1.02	−0.42 ± 1.08	.264
Successful eradication	n = 56	n = 12	
*SEPTIN9* (logPMR)	0.81 ± 0.42	1.05 ± 0.45	.081
*SDC2* (logPMR)	0.76 ± 0.40	0.98 ± 0.41	.090
Combined methylation score (*Z*-score)	0.02 ± 1.13	0.58 ± 1.16	.126
Persistent positive	n = 54	n = 18	
*SEPTIN9* (logPMR)	0.99 ± 0.45	1.42 ± 0.46	<.001
*SDC2* (logPMR)	0.91 ± 0.42	1.31 ± 0.40	<.001
Combined methylation score (*Z*-score)	0.41 ± 1.09	1.24 ± 1.02	.006

PMR, percentage of methylated reference.

## Data Availability

The data that support the findings of this study are available on request from the corresponding author.
